# Editorial: Integrating nature-based solutions for land degradation neutrality and deriving co-benefits

**DOI:** 10.3389/fpls.2026.1766472

**Published:** 2026-01-29

**Authors:** Ting Hua, Yi Han, Yang Yu, Miguel Inácio, Paulo Pereira

**Affiliations:** 1 Industrial Ecology Programme, Department for Energy and Process Engineering, Norwegian University of Science and Technology, Trondheim, Norway; 2 Key Laboratory of Poyang Lake Wetland and Watershed Research (Ministry of Education), School of Geography and Environment, Jiangxi Normal University, Nanchang, China; 3 School of Soil and Water Conservation, Beijing Forestry University, Beijing, China; 4 Department of Agricultural Sciences, Institute of Agronomy, University of Natural Resources and Life Sciences, Vienna, Austria; 5 Environmental Management Research Laboratory, Mykolas Romeris University, Vilnius, Lithuania

**Keywords:** climate change, ecological restoration, ecosystem service, land degradation neutrality (LDN), nature-based solution

Land degradation remains one of the most significant challenges of the 21st century, undermining ecosystem integrity, agricultural productivity, biodiversity, and human well-being (Prăvălie, 2021). Global economic losses associated with land degradation, arising from declines in multiple ecosystem services, could total 6.3–10.6 trillion US dollars annually (UNCCD, 2017). In response to growing policy momentum and scientific consensus, Land Degradation Neutrality (LDN) has gained prominence as a unifying global objective to achieve ‘zero net land degradation’ (UNCCD, 2015). In parallel, Nature-based Solutions (NbS), actions that protect, restore, and sustainably manage natural or modified ecosystems, have attracted increasing attention as a practical approach toward LDN, while delivering substantial environmental and social co-benefits (IUCN, 2016). However, the effectiveness of NbS is shaped by complex interactions among intervention types, biophysical conditions, climate, and management practices, resulting in substantial heterogeneity in ecosystem service provision across different contexts (Griscom et al., 2017; Seddon et al., 2020). Multidisciplinary approaches and governance perspectives could strengthen both micro- and macro-level understanding of how NbS can most effectively support LDN and other co-benefits (Griscom et al., 2017; Seddon et al., 2020). Against this backdrop, this Research Topic of *Frontiers in Plant Science* features 11 papers that span forests, grasslands, croplands, coastal ecosystems, and urban landscapes. Collectively, they demonstrate the potential of NbS to mitigate and reverse land degradation, while navigating complex interactions among climate change, hydrological processes, soil dynamics, and social constraints.

## Diagnosing degradation risks and climate pressures as a foundation for NbS

Several papers in this Research Topic provided critical diagnostics that help shape effective NbS strategies to mitigate degradation risks and guide restoration efforts. Rui et al. quantified the joint effects of climate change and human activities on grassland growth in Xinjiang, showing that vegetation greening is mainly attributable to a warming, humidifying climate. In contrast, local plant loss is more strongly associated with human pressures such as overgrazing and urbanisation. Hai et al. investigated the mechanisms by which precipitation frequency and amplitude influence forest growth in Northeast China, identified degradation hotspots in areas with high-frequency but low-amplitude fluctuations, and provided key climate-ecological insights for NbS-informed forest management. Zhu et al. compared multiple deep soil moisture profiles on the Loess Plateau and demonstrated that long-term artificial planting can substantially deplete deep soil water relative to farmland and native grassland, highlighting the hydrological trade-offs of restoration in water-scarce regions.

In cropland landscapes, Liao et al. mapped cultivated land degradation in southern China and disentangled the relative contributions of natural factors and management interventions (e.g., straw incorporation, fertilisation). The authors then translate these diagnostics into targeted restoration options, including deep-rooted crops, crop rotations and straw incorporation. In rapidly urbanising regions, Liu et al. analysed invasive alien plant species distributions across urban green spaces, rural areas and croplands, showing that native plant diversity and socio-economic conditions jointly shape invasion patterns and underscoring the need for differentiated NbS approaches in urban and peri-urban landscapes. Together, these studies highlight robust diagnostics of degradation and associated abiotic and biotic pressures to guide the design of NbS and support avoiding unintended consequences.

## NbS for enhancing ecosystem functioning and services in diverse environments

A second group of contributions focused on how NbS interventions can stabilise ecosystem functioning and enhance ecosystem multifunctionality, such as soil quality and carbon stocks. In an ecologically vulnerable region of the Taihang Mountains, Zhang et al. demonstrated, across 72 sites, that land-use transition (from barren hills to cropland to pear orchards) combined with intercropping (ryegrass and winter rape) can substantially increase soil carbon and nutrient levels. That is because cover crops and root systems increase carbon input, optimise soil structure, and form a nutrient-carbon pool synergy. On the Qinghai–Tibet Plateau, Li et al. investigated long-term cultivation of Kentucky bluegrass as both a restoration intervention and alternative forage species, improving soil fertility and microbial community structure over time, thereby informing sustainable management practices to support soil health and ecosystem stabilisation.

Complementing these soil-focused studies, Xue et al. found that simulated warming and litter removal in alpine grassland generate opposite above- and below-ground responses. Warming reduces vegetation cover and above-ground multifunctionality while enhancing below-ground functions. In contrast, litter removal partly buffers the warming-induced declines. Zhan et al.extended ecological process understanding to management by developing zoning-based adaptive strategies for Tibetan Plateau grasslands using the “quality–pressure–resilience” framework. Their approach delineates zones for protection, selective enhancement and active intervention, thereby providing an illustrative roadmap for conserving and restoring alpine grassland.

Beyond terrestrial systems, this Research Topic also expands the NbS focus to coastal ecosystems. Marletta et al. evaluated the use of different growth promoters in seagrass (*Cymodocea nodosa*) restoration, comparing synthetic plant growth regulators with plant growth-promoting bacteria in controlled aquarium experiments. This study showed that applying plant growth-promoting bacteria markedly improves survival and vegetative propagation, whereas synthetic hormones provide only limited additional benefits. Collectively, these studies demonstrate the diverse mechanisms through which NbS can reinforce ecosystem functioning and multifunctionality in both terrestrial and coastal environments, delivering actionable evidence to support more resilient management.

## Social and biophysical constraints on scaling NbS

Finally, the Topic included contributions that reflect on the multiple biophysical and social constraints for NbS deployment. Zha and Zhang synthesised diverse empirical evidence on the mitigation and adaptation benefits of agroforestry, while emphasising that biophysical and social constraints strongly shape both its deployment and performance. Their perspective aligns with recent research pointing to constraints on NbS deployment, posed by biodiversity conservation and water scarcity (Gvein et al., 2023); the influences of carbon pricing on NbS economic feasibility (Lu et al., 2022); and competition with demands for food, timber, and bioenergy production (Fuhrman et al., 2020). Overcoming these challenges will require coordinated, multi-level engagement involving farm-level practitioners, policymakers, and a broad set of stakeholders (Pereira and Zhao, 2025).

## Concluding remarks

Together, these papers in this Research Topic provide practical evidence on when, where and how NbS can support long-term benefits across the land-water-climate-people nexus. Such insights are particularly timely, as decision-makers urgently need to understand the role that NbS can play in advancing LDN and limiting further global temperature increases. These papers also highlight the necessity of multidisciplinary approaches—spanning ecology, remote sensing, agronomy, economics, and the social sciences—to design effective and context-appropriate interventions. Future research will need to build on this foundation by integrating long-term monitoring, co-designed interventions with local stakeholders, and comparative analyses across regions and ecosystem types, so that NbS can more reliably support progress toward LDN and broader co-benefits.
